# Mild Effect of Nalmefene on Alcoholic Cue-Induced Response Invigoration in Alcohol Use Disorder Without Accompanying Changes in Electrophysiological Signatures of Early Visual Processing and Executive Control

**DOI:** 10.3389/fphar.2019.01087

**Published:** 2019-09-26

**Authors:** Bernadett I. Gál, Tünde Kilencz, Anita Albert, Ildikó Demeter, Klára Mária Hegedűs, Zoltán Janka, Gábor Csifcsák, Péter Z. Álmos

**Affiliations:** ^1^Department of Psychiatry, Faculty of Medicine, University of Szeged, Szeged, Hungary; ^2^Department of Psychiatry and Psychotherapy, Semmelweis University, Budapest, Hungary; ^3^Department of Cognitive and Neuropsychology, Institute of Psychology, Faculty of Humanities and Social Sciences, University of Szeged, Szeged, Hungary; ^4^Department of Psychology, Faculty of Health Sciences, UiT The Arctic University of Norway, Tromsø, Norway; ^5^Department of Psychiatry, Psychosomatics and Psychotherapy, Center of Mental Health, University Hospital of Würzburg, Würzburg, Germany

**Keywords:** nalmefene, alcohol use disorder, response inhibition, incentive salience, event-related potentials, Go/NoGo task

## Abstract

Nalmefene is approved for as-needed pharmacological treatment in alcohol use disorder (AUD) by the European Medicines Agency. While the cellular effects of nalmefene have been thoroughly investigated, data are very limited on how this agent influences neural signals associated with inhibitory control and the visual analysis of environmental cues. This double-blind crossover study assessed the behavioral and neural effects of acute nalmefene administration in patients diagnosed with AUD. In experiment 1, we validated our experimental paradigm (electroencephalography combined with a modified Go/NoGo task using images of alcoholic and nonalcoholic drinks as prime stimuli) in 20 healthy adults to ensure that our protocol is suitable for assessing the behavioral and neural aspects of executive control. In experiment 2, we recruited 19 patients with AUD, and in a double-blind crossover design, we investigated the effects of nalmefene versus placebo on task performance (response accuracy, the sensitivity index, and reaction times), visual responses to appetitive cues (occipital P1, N1, and P2 components), and electrophysiological markers of conflict detection and response inhibition (frontal N2 and P3 waveforms). Under placebo, patients produced faster reaction times to alcohol-primed Go stimuli, an effect that was weak despite being statistically significant. However, the effect of alcoholic cues on the speed of response initiation disappeared after receiving nalmefene. We found no placebo versus nalmefene difference regarding our patients’ ability to accurately inhibit responses to NoGo stimuli or for occipital and frontal event-related potentials. Our results suggest that nalmefene might be potent in reducing the vigor to act upon alcoholic cues in AUD patients, but this effect is most probably mediated *via* subcortical (rather than cortical) neural circuits.

## Introduction

There is a considerable debate regarding nalmefene, an opioid agent, which has been approved by the European Medicines Agency (European Medicines Agency, 2013) as a treatment option in alcohol use disorder (AUD) (Gual et al., 2013). According to a meta-analysis, the drug is able to improve behavioral outcomes in patients with AUD (Mann et al., 2016), while others show that it has a limited efficacy in alcohol dependence therapy (Palpacuer et al., 2015; Soyka and Müller, 2017), and rather, it is more useful as an as-needed, harm-reducing pharmacotherapy (European Medicines Agency, 2013; Aubin et al., 2015; Korpi et al., 2016). Furthermore, Foo et al. (2019) demonstrated recently in an animal model that the effectiveness of nalmefene is influenced by individual drinking profiles. This contradiction may root in a significant information gap regarding how molecular and system-wide properties of the drug are connected, e.g., what is nalmefene’s effect on cortical correlates of information processing.

The reinforcing mechanism of alcohol use is influenced by the interplay of opioid and dopaminergic systems (Spreckelmeyer et al., 2011). Alcohol modulates dopamine concentrations in the mesolimbic system in a dose-dependent manner, an effect that is mediated *via* opioid receptors, among other molecular mechanisms (Boileau et al., 2003; Martinez et al., 2005; Soyka et al., 2017). Through enhancing dopamine release, repeated alcohol consumption can alter reward prediction error signals in the midbrain and ventral striatum, which in turn will lead to maladaptive learning and to the development of rigid and habitual alcohol-consuming responses (Redish, 2004; Everitt and Robbins, 2005). The transition from goal-directed to habitual drug use is heavily influenced by the Pavlovian learning system that conditions the individual to environmental cues (e.g., the sight of one’s favorite pub) associated with reward, and facilitates response tendencies towards such stimuli, resulting in drug-seeking and drug-consuming behavior (Everitt and Robbins, 2005; Garbusow et al., 2014). By its direct effects on mu- and kappa opioid receptors, nalmefene probably decreases ethanol consumption by modulating dopamine transmission *via* GABAergic interneurons in the ventral striatum (Rose et al., 2016). Thus, *via* its effect on opioid and dopaminergic neurotransmission, nalmefene has a potential to reduce alcohol consumption by dampening the impact of Pavlovian responses on choice behavior, that is, by reducing the incentive salience of environmental cues.

In the systematic review of Pujol et al. (2018), the authors explored the cognitive effects of pharmacotherapy in addiction, but they pointed out the lack of clinical trials on the cognitive impact of nalmefene in patients with AUD. Only one study tried to fill this informational gap with investigating the behavioral and neural effects of nalmefene in 18 nontreatment seeking alcohol-dependent patients (Quelch et al., 2017). The authors found that nalmefene prolonged reaction times and reduced the amount of earned reward in a monetary reward anticipation task while keeping overall response accuracy unchanged. In addition, blood-oxygen-level-dependent signals assessed with functional magnetic resonance imaging were reduced following nalmefene treatment in the dorsal striatum, putamen, globus pallidus, brainstem, and the cerebellum. This result is in line with the above discussed molecular effects of nalmefene on the mesolimbic dopaminergic system. However, it remains unknown whether nalmefene also influences cortical mechanisms associated with cognitive control and decision making.

The answer to this question might have important therapeutic implications since behavioral choices and response inhibition were shown to be altered after acute alcohol intoxication (Fillmore et al., 2008) and in individuals with drinking problems (Ames et al., 2014) or AUD (Schad et al., 2019). Importantly, detoxified patients remaining abstinent during the follow-up period of 6 months demonstrated reduced responding to emotionally neutral stimuli embedded in an alcohol-related background, accompanied by increased hemodynamic responses in the right nucleus accumbens (Schad et al., 2019). These effects were absent in relapsers, indicating that stronger inhibitory control in the presence of alcoholic cues is related to the ability to maintain abstinence in AUD. However, Schad et al. (2019) did not find evidence for the involvement of any cortical area in cue-associated response inhibition, rendering the link between the prefrontal cortex and cognitive control over alcoholic cues in AUD speculative.

Utilization of event-related potentials (ERPs) paired with cognitive tasks is a frequently used method to investigate executive processes such as conflict detection, inhibitory control, and response selection (Kok et al., 2004). In the Go/NoGo task, subjects have to inhibit their responses when a rarely encountered NoGo stimulus is presented, whereas they have to make fast motor responses to frequently presented Go stimuli (Oddy and Barry, 2009; Hong et al., 2017). NoGo signals typically elicit two large ERPs above frontocentral scalp areas: the N2 is a negative waveform peaking between 200 and 300 ms after stimulus onset, while the subsequent P3 is a positive-going component arising 300–500 ms after stimulus presentation. The N2 has been primarily associated with conflict monitoring, whereas the P3 was implicated in response inhibition (Huster et al., 2011; Luijten et al., 2014). These components reflect activity of a network consisting of several areas, including the dorsal anterior cingulate cortex (dACC), basal ganglia, and the presupplementary motor cortex (Huster et al., 2010; Huster et al., 2011). Given that the N2/P3 complex is a reliable neurophysiological marker of conflict detection and top–down inhibitory control, these waveforms can be used to evaluate whether nalmefene acts on executive process during a Go/NoGo task.

Heightened salience to alcohol- and other drug-related cues is related to attentional and emotional modulation of sensory processing (Volkow et al., 2008; Anderson, 2016). Early visual ERPs like the posterior P1, N1, and P2 components (peaking at 90–110, 120–150, and 160–190 ms poststimulus, respectively) are enhanced in amplitude when attention is directed towards visual stimuli (Clark and Hillyard, 1996; Han et al., 2005) or when stimuli have strong emotional value (Vuilleumier, 2005). Thus, by assessing the effects of nalmefene on these waveforms during the presentation of alcoholic and nonalcoholic cues, we can investigate if this agent affects early sensory analysis of salient environmental stimuli.

In the current study, we aimed to assess how nalmefene influences sensory processing of appetitive cues and executive control related to response inhibition in alcohol-dependent patients. We used a modified Go/NoGo task in which emotionally neutral Go and NoGo stimuli were preceded by alcoholic and nonalcoholic cues, presented in the background. We chose this paradigm because it not only allows to investigate the effect of executive control over emotionally neutral visual stimuli but also to test the influence of appetitive contextual triggers on motor responses or the suppression thereof. In this respect, our task bears resemblance to Pavlovian-to-instrumental transfer (PIT) tasks that are sensitive to the interaction of instrumental and Pavlovian learning systems and hence can be used to assess value-based learning mechanisms and decision-making strategies in psychiatric conditions such as AUD (Garbusow et al., 2014).

In experiment 1, we validated our experimental paradigm in a group of 20 healthy adults. This was done because, as the first experiment of this kind in our lab, we wanted to make sure that the well-documented Go versus NoGo effect on frontal N2 and P3 waveforms can be replicated with our modified, cue-primed protocol. Moreover, we also wanted to ensure that alcoholic cues do not alter behavioral and neural responses in healthy controls because we anticipated such effects to emerge in patients under placebo only. To match the design of experiment 2 as well as possible, we administered placebo pills to all participants in an open-label fashion. In experiment 2, we recruited short-term abstinent patients diagnosed with AUD, and in a double-blind crossover study, we evaluated the effects of nalmefene versus placebo on behavioral measures and ERP correlates of early visual processing and conflict monitoring/response inhibition.

Based on the established clinical efficacy of nalmefene, we expected that active treatment would improve inhibitory control in patients relative to placebo, manifesting in better response accuracy and higher sensitivity (*d*′) to refrain from responding to NoGo stimuli, especially when these stimuli are primed with images of alcoholic drinks. We also anticipated that patients receiving placebo would exhibit faster reaction times to alcohol-primed Go stimuli, indicative of enhanced response vigor to appetitive stimuli, and that this effect will be normalized following nalmefene administration. We hypothesized that, if the beneficial effect of nalmefene is due to more efficient top–down cognitive control, it would be associated with increased NoGo-associated frontal N2/P3 amplitudes, pointing towards more efficient conflict detection/response inhibition after active treatment. Conversely, if nalmefene dampened the emotional salience of alcoholic cues during the course of early visual processing, we expected to observe reduced early visual ERPs elicited by alcoholic relative to nonalcoholic cues. Finally, we did not exclude the possibility that nalmefene would influence task performance only, without modulating any ERP component. This result would indicate that nalmefene primarily acts on subcortical structures such as the striatal region (Quelch et al., 2017), without robustly influencing frontal and occipital cortical processes in our task.

## Experiment 1

### Methods and Materials

#### Participants

We enrolled 20 healthy adults to validate our experimental paradigm (**Table 1**). Mental and neurological disorders and alcohol and drug dependence were exclusion criteria (based on self-report). With respect to the frequency of alcohol consumption, eight participants reported no alcohol consumption at all, nine consumed alcoholic drinks on one to three occasions monthly, and three consumed alcohol once per week. Participants received payment for participation (5,000 Hungarian Forints corresponding to approximately 15 Euros). Every participant was informed about the study procedure, and they declared their agreement in a written consent. The study received certificate of ethical approval from Clinical Research Coordination Centre, University of Szeged (number of ethical approval: 171/2014).

**Table 1:** Demographic and clinical characteristics of participants.

Characteristics	Healthy group (experiment 1)	Patients (experiment 2)
**Group size**	20	19
**Sex (female/male)**	5/15	5/14
**Age (years)**	43.70 ± 6.61	48.32 ± 10.61
**Education (levels 1/2/3)**	0/12/8	3/9/7
**Frequency of alcohol consumption**	1. No alcohol consumption at all (*N* = 8) 2. Consumed alcohol consumption one to three occasions monthly (*N* = 9) 3. Consumed alcohol consumption once per week (*N* = 3)	Short-term abstinent group (<2 months)
**Duration of problematic alcohol consumption (years)**	–	15.68 ± 11.87
**Duration of lifetime abstinence (years)**	–	2.16 ± 2.81

Data are mean ± SD or N. Educational levels: 1, primary education or below; 2, secondary education (high school, vocational school); 3, postsecondary education (college, graduate school).

#### Study Design and Task Description

**Figure 1:**
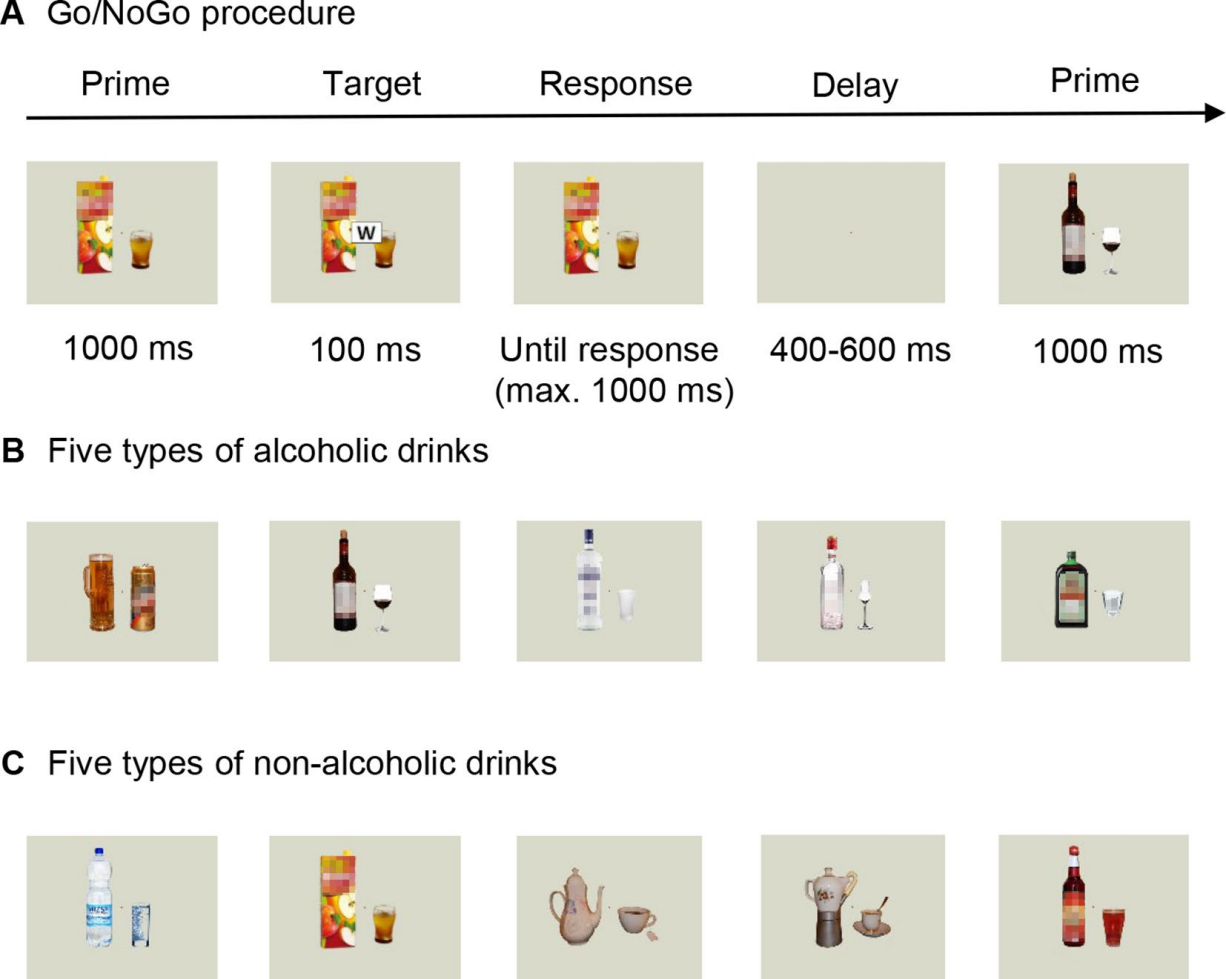
Overview of our behavioral task. **(A)** Trial sequence in the Go/NoGo paradigm, with pictures of either **(B)** alcoholic drinks (beer, wine, vodka, fruit brandy, and liqueur) or **(C)** nonalcoholic beverages (mineral water, apple juice, tea, coffee, raspberry syrup) as prime stimuli. Please note that colored stimuli were used in both experiments. The blurred effect on the images was applied only in the publication.

Since we initially planned to compare the results from this group to those obtained from AUD patients after receiving either nalmefene or placebo, we asked control participants to take our placebo product (vitamin D3; dose, 25 μg) before the start of the data collection. Every participant was aware of receiving placebo in the experiment, which is one reason for why we did not compare data from the two groups eventually (see: “Limitations”). We used a special computerized Go/NoGo task combined with electroencephalography (EEG) to assess the behavioral and neural correlates of the processing of alcoholic stimuli and their influence on executive control. The task consisted of stimuli depicting alcoholic drinks or alcohol-free beverages as primes (Petit et al., 2012; Kreusch et al., 2014, **Figure 1**). Participants were sitting in a dark room, in front of a 19″ cathode ray tube monitor with a viewing distance of 80 cm. Stimuli were presented, and responses were recorded with E-prime 2.0 Professional program (Psychology Software Tools, Inc., Sharpsburg, Pennsylvania, USA). During the task, 50% of the participants had to press the space bar when they saw one letter (targets: W or M) appearing in the middle of the prime picture (Go stimulus) and had to inhibit responding for the other letter (NoGo stimulus). Stimuli were counterbalanced across patients. Participants had to accomplish the task as fast and accurate as possible. At the beginning of a trial, alcoholic and neutral prime pictures were shown for 1,000 ms, followed by a target stimulus, presented for 100 ms. An additional 1,000 ms was available for responding, and the trial ended earlier if a response was given. Trials were separated by a screen containing a white fixation cross (presented for a random period between 400 and 600 ms) (**Figure 1**). The test contained 600 stimuli divided into 10 blocks, separated by short breaks. Primes either depicted one of the five types of alcoholic (beer, wine, vodka, fruit brandy, and liqueur) or nonalcoholic (water, apple juice, tea, coffee, raspberry syrup) drinks. Since our primes were asymmetric stimuli (**Figure 1**), every image was presented either in its original or mirrored version in 50–50% of trials. The experiment began with a short practice block consisting of 20 stimuli. Each block contained 60 trials, with 20 alcohol-primed Go stimuli, 20 nonalcohol-primed Go stimuli, 10 alcohol-primed NoGo stimuli, and 10 nonalcohol-primed NoGo stimuli, presented in a random order. The whole procedure lasted for 30–40 min.

#### EEG Recording and Analysis

EEG was collected with a 32-channel Biosemi EEG System (BioSemi B.V., Amsterdam, The Netherlands). Sampling rate was set to 1,024 Hz; no frequency filters were used during data collection. Ag/AgCl electrodes were attached to an elastic EEG cap, with electrodes positioned in accordance with the International 10-20 System (Jasper, 1958). Recording sites were Fp1, Fp2, F3, F4, F7, F8, Fz, FC1, FC2, FC5, FC6, C3, C4, Cz, T7, T8, CP1, CP2, CP5, CP6, P3, P4, P7, P8, Pz, O1, O2, Iz, TP9, and TP10. The reference and ground electrodes (common mode sense and driven right leg electrodes in the active two system; Metting van Rijn et al., 1990) were placed in close proximity to the Cz position.

The data were analyzed with the Brain Vision Analyzer 2.0.3 software (Brain Products GmbH, Gilching, Germany). Data were re-referenced to common average and filtered with 0.5 Hz high-pass zero phase-shift Butterworth filter (24 dB/oct). Ocular artifacts were removed using an algorithm described by Gratton, Coles, and Donchin (Gratton et al., 1983). Continuous EEG was segmented to epochs from 100 ms prestimulus to 600 ms poststimulus and baseline corrected from −100 to 0 ms. Artifact rejection was conducted semiautomatically, so epochs were visually also inspected for horizontal eye movement- or muscle activation-related artifacts. After averaging, data were filtered with a 30-Hz (occipital responses to prime stimuli) or 15-Hz (frontal ERPs evoked by Go/NoGo stimuli) low-pass zero phase-shift Butterworth filter (24 dB/oct).

Visual P1, N1, and P2 ERPs were analyzed at the pooled occipital O1/O2 channels. Mean amplitudes of each component were measured in a 40-ms time window, centered around the group-averaged peak of waveforms collapsed across all experimental conditions (P1: 120 ms; N1: 180 ms; P2: 260 ms). The frontal N2 was measured at electrode Fz, whereas the subsequent P3 was quantified at electrode Cz because of its more central scalp distribution. Mean N2 and P3 amplitudes were extracted from a 40-ms time window centered at 290 and 420 ms, respectively.

#### Statistical Analysis

All statistical analyses were performed with SPSS for Windows, Version 22.0 (IBM SPSS Statistics, Armonk, New York State, USA). Accuracy was determined as the proportion of correct responses to each trial type (alcoholic vs. nonalcoholic primes; Go vs. NoGo stimuli). We used hit rates (proportion of correct responses to Go stimuli) and false alarms (proportion of motor responses to NoGo stimuli) from alcohol- and nonalcohol-primed trials to calculate the sensitivity index (*d*′) from signal detection theory. This was done using the following formula: *d*′ = *Z*_hit rate_ − *Z*_false alarm rate_, where *Z* is the inverse of the normal distribution function (Macmillan and Creelman, 2004). This parameter represents the individual’s efficacy of decision making (with higher values corresponding to more accurate responding) and has been widely used in Go/NoGo tasks in studies involving AUD patients (Saunders et al., 2008; Ames et al., 2014). In addition, we calculated median reaction times (RTs) for Go stimuli, separately for both priming conditions. Owing to near-ceiling effects in terms of response accuracy, we could not analyze RTs for incorrect responses to NoGo stimuli. Behavioral outcomes were analyzed with nonparametric tests because the assumption of normality was violated for all measures (Shapiro–Wilk test, accuracy: *p* < 0.001; *d*′: *p* = 0.047; RT: *p* = 0.008). In the case of response accuracy, variation across the four trial types were assessed using Friedman test, with Wilcoxon signed-rank tests for follow-up pairwise comparisons using Bonferroni-adjusted alpha levels of 0.0125. For *d*′ and RT, potential differences in performance between trials with alcoholic versus nonalcoholic primes were assessed with Wilcoxon signed-rank tests at an alpha level of 0.05.

Mean ERP amplitudes were analyzed with repeated-measures analysis of variance (ANOVA) with target (Go or NoGo; for frontal ERPs only) and prime (alcoholic or nonalcoholic; for occipital and frontal ERPs) as within-subject factors. Tests with *p* < 0.05 were considered as significant. Significant interactions were further evaluated with Bonferroni-corrected post-hoc tests. In order to demonstrate the magnitude of the observed effects, partial eta-squared (ခη_p_^2^) values are also shown.

### Results

#### Behavioral Measures

**Figure 2:**
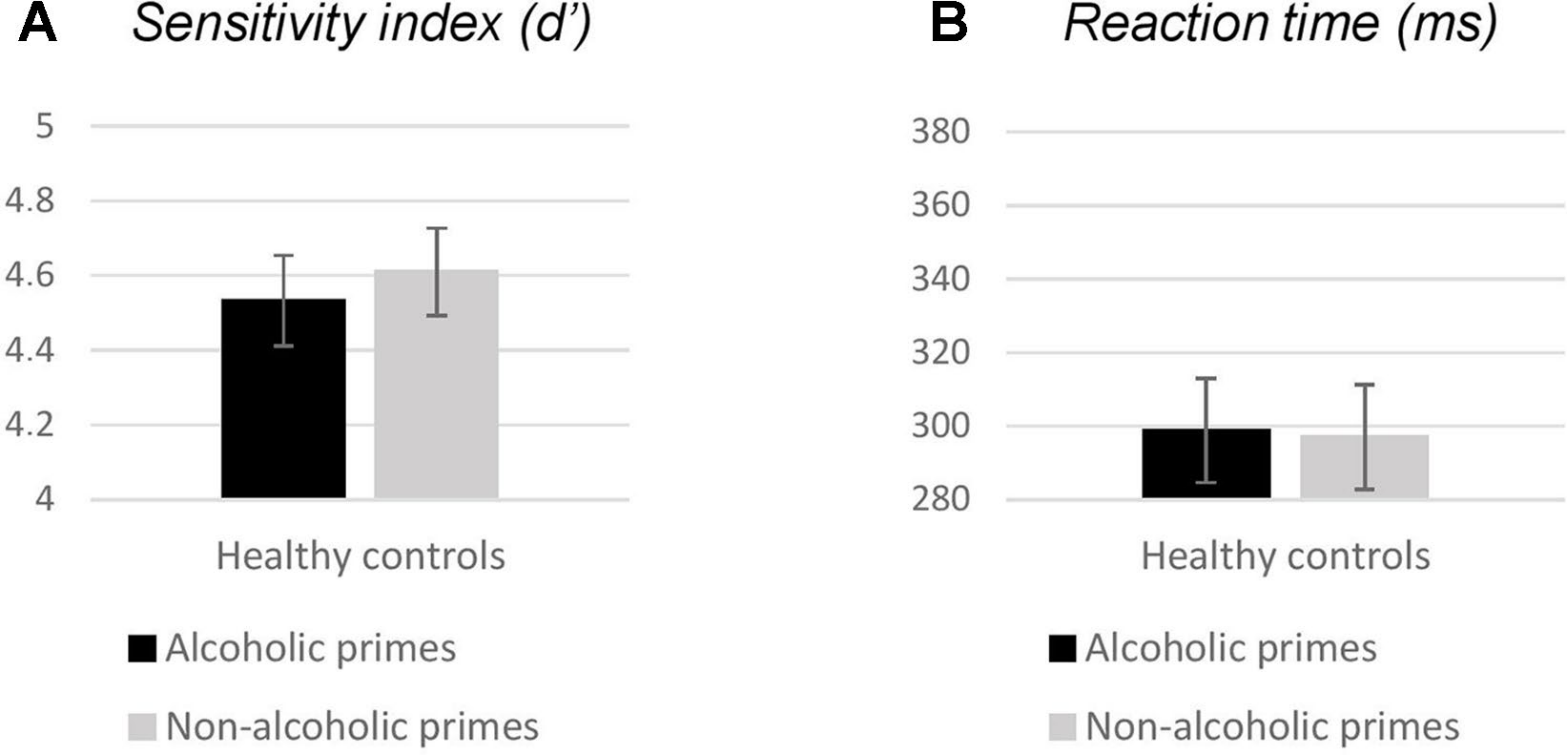
Behavioral measures (**A**: sensitivity index, **B**: reaction times) obtained in experiment 1. Bars represent mean; error bars depict standard errors. Reaction times were calculated for Go responses only.

We found a significant variation in response accuracy across trial types [χ^2^(3) = 35.77; *p* < 0.001]. Follow-up pairwise comparisons revealed no effect of prime (Go: *Z* = −0.33, *p* = 0.739; NoGo: *Z* = −1.64, *p* = 0.100), but significant differences between performance for Go versus NoGo trials (alcoholic primes: *Z* = −3.71, *p* < 0.001; nonalcoholic primes: *Z* = −3.30, *p* = 0.001; **Table 2**), with more accurate responding to Go stimuli. Neither *d*′ nor RT was significantly affected by prime type (*d*′: *Z* = −0.69, *p* = 0.490, **Figure 2A**; RT: *Z* = −0.72, *p* = 0.468, **Figure 2B**). Overall, these results indicate that images depicting alcoholic beverages did not interfere with executive control in our healthy group.

**Table 2:** Behavioral accuracy (proportion of correct responses) in the four trial types from both experiments (mean ± standard deviations).

	Alcoholic primes		Nonalcoholic primes	
	Go trials (hit rates)	NoGo trials (1—false alarm rates)	Go trials (hit rates)	NoGo trials (1—false alarm rates)
**Healthy group** **(experiment 1)**	0.9975 ± 0.005	0.9470 ± 0.059	0.9980 ± 0.005	0.9615 ± 0.046
**Patients—placebo session** **(experiment 2)**	0.9968 ± 0.006	0.9447 ± 0.047	0.9958 ± 0.08	0.9547 ± 0.05
**Patients—nalmefene session** **(experiment 2)**	0.9937 ± 0.013	0.9274 ± 0.073	0.9916 ± 0.018	0.9305 ± 0.082

#### Event-Related Potentials

##### Occipital P1, N1, and P2 amplitudes

**Figure 3:**
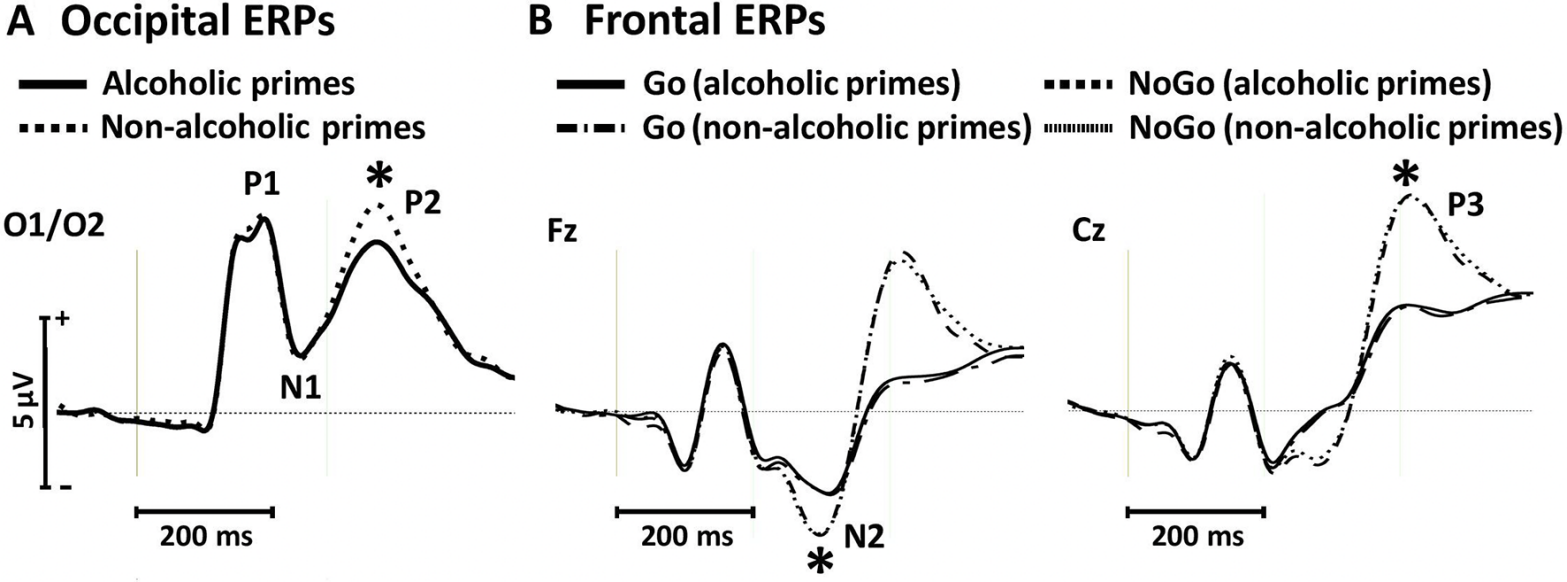
Occipital event-related potentials evoked by alcoholic and nonalcoholic primes **(A)** and frontal event-related potentials evoked by Go and NoGo targets in the context of alcoholic and nonalcoholic primes **(B)** in experiment 1. Stars depict significant (*p* < 0.05) differences between amplitudes obtained in different conditions.

For the P1 and N1 amplitudes, we found no significant main effects of prime [P1: *F*(1, 19) = 1.33, *p* = 0.262, η_p_^2^ = 0.066; N1: *F*(1, 19) = 0.05, *p* = 0.811, η_p_^2^ = 0.003; **Figure 3A**]. However, the P2 component was significantly larger for nonalcoholic primes [*F*(1, 19) = 48.84, *p* < 0.001, η_p_^2^ = 0.720; **Figure 3A**].

##### Frontal N2 and P3 amplitudes

We found larger N2 amplitudes in NoGo relative to Go trials [*F*(1, 19) = 16.23, *p* = 0.001, η_p_^2^ = 0.461; **Figure 3B**]. Neither the main effect of prime [*F*(1, 19) = 0.03, *p* = 0.858, η_p_^2^ = 0.002], nor the target × prime interaction was significant [*F*(1, 19) = 0.002, *p* = 0.967, η_p_^2^ < 0.001]. Similar effects were observed for the P3 component, with larger amplitudes for NoGo stimuli [*F*(1, 19) = 72.30, *p* < 0.001, η_p_^2^ = 0.792; **Figure 3B**] but no main effect of prime [*F*(1, 19) = 0.33, *p* = 0.571, η_p_^2^ = 0.017] or target × prime interaction [*F*(1, 19) = 0.005, *p* = 0.946, η_p_^2^ < 0.001]. These findings support behavioral results of the absence of prime-associated effects on neural correlates of conflict detection and response inhibition in the control group.

## Experiment 2

### Methods and Materials

#### Participants

We enrolled 20 short-term (< 2 months) abstinent patients diagnosed with AUD (World Health Organization, 1992) at the Department of Psychiatry, University of Szeged, Hungary. Main demographical characteristics, alcohol consumption, and abstinence history of all patients are presented in **Table 1**. Comorbid mental and neurological disorders (verified by anamnestic documentation) and acute withdrawal symptoms were exclusion criteria. All patients were tested with the Hungarian version of the Wechsler Adult Intelligence Scale (Rózsa et al., 2010a; Rózsa et al., 2010b) to screen for extreme low IQ. We excluded one patient because his IQ score was in the zone of mental retardation (full-scale intelligence quotient = 60), whereas scores for other patients were in the normal range (*M* = 94.26, SD = 17.29, minimum = 70, maximum = 125). Thus, data from 19 patients were included in the final analysis. Our study received certificate of ethical approval from Clinical Research Coordination Centre, University of Szeged (number of ethical approval: 171/2014). Every participant was informed about the study procedure and about possible adverse reactions of nalmefene, and they declared their agreement by signing the informed consent form.

#### Study Design and Task Description

In a double-blind, placebo-controlled crossover study design, we administered either placebo (vitamin D3, 25 μg) or nalmefene (18 mg) orally, in forms of identical white and tasteless pills of oval shape. We decided to investigate the effects of a single dose of nalmefene because this agent is recommended to be administered in a single dose on days when patients perceive a risk of consuming alcohol (European Medicines Agency, 2013). We verified that none of the patients took nalmefene or our vitamin D3 product earlier. Given that nalmefene reaches its peak plasma concentration in 1.5 h following oral intake, pills were administered ∼1 h before the start of preparation for data collection. In order to control for potential learning effects in the Go/NoGo procedure, 50% of the participants received nalmefene first. On average, 39.7 h has passed between the repeated times of testing (min. 20 h, max. 149 h). We note that patients reported side effects after receiving nalmefene only. These were always of mild-to-moderate intensity: fatigue (*N* = 3), dejection (*N* = 2), dizziness (*N* = 2), nausea (*N* = 1), headache (*N* = 1), and dry mouth (*N* = 1). The Go/NoGo task was identical to that of experiment 1.

#### EEG Recording and Analysis

We used a 32-channel Nicolet Bravo Multimodal EEG System (EMS Co, Korneuburg, Austria) for recording neural activity in experiment 2 because the EEG system used in the first experiment was not available anymore. Sampling rate was set to 1,024 Hz; no frequency filters were used during data collection. Ag/AgCl electrodes were attached to an elastic EEG cap, with electrodes positioned in accordance with the International 10-20 System (Jasper, 1958). Electrodes were placed at scalp sites Fp1, Fp2, F3, F4, F7, F8, Fz, FC1, FC2, FC5, FC6, C3, C4, Cz, T7, T8, CP1, CP2, CP5, CP6, P3, P4, P7, P8, Pz, O1, O2, Oz, TP9, and TP10. Electrode AFz was used as reference, and electrode Fpz served as ground. The procedure of the data analysis was the same as described in experiment 1.

#### Statistical Analysis

As for behavioral data collected in experiment 1, the assumption of normality was violated for all three measures (Kolmogorov–Smirnov test with Lilliefors correction, accuracy: *p* < 0.001; *d*′: *p* = 0.007; RT: *p* = 0.036). The nonparametric Friedman test was used to assess if there was a significant variation across treatment conditions and trial types. Follow-up pairwise comparisons were performed with Wilcoxon signed-rank tests, using Bonferroni-adjusted alpha levels of 0.004 (accuracy) or 0.0125 (*d*′ and RT).

### Results

#### Behavioral Measures

There was a significant variability in response accuracies across the four trial types in the two treatment sessions [χ^2^(7) = 92.42; *p* < 0.001, **Table 2**]. Pairwise comparisons indicated no effect of prime (*Z* > −1.42, *p* > 0.156) or treatment (*Z* > −1.89, *p* > 0.058) but significantly better accuracies in Go relative to NoGo trials (nalmefene + alcoholic prime: *Z* = −3.73, *p* < 0.001; nalmefene + nonalcoholic prime: *Z* = −3.24, *p* = 0.001; placebo + alcoholic prime: *Z* = −3.83, *p* < 0.001; placebo + nonalcoholic prime: *Z* = −3.64, *p* < 0.001).

**Figure 4:**
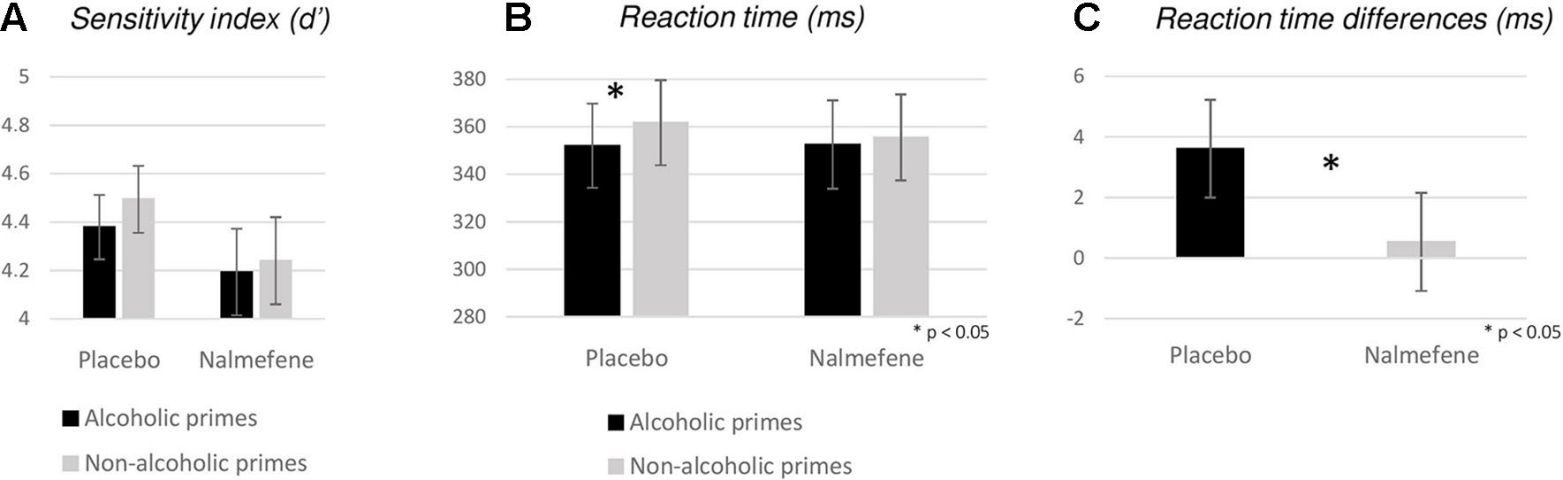
Behavioral measures (**A**: sensitivity index, **B**: reaction times, **C**: reaction time differences for nonalcoholic–alcoholic primes) obtained for experiment 2. Bars represent mean; error bars depict standard errors. Reaction times were calculated for Go responses only. Stars depict significant (*p* < 0.05) disparities between reaction times and differences of reaction times obtained in different priming and grouping conditions.

Although we also found a significant effect for *d*′ [χ^2^(3) = 8.71; *p* = 0.033], follow-up pairwise comparisons did not reveal significant effects at our corrected alpha level (*Z* > −2.06, *p* > 0.038). We note, however, that the sensitivity index was numerically smaller following nalmefene treatment for both prime types (**Figure 4A**).

With respect to RTs, we found significant variability across experimental conditions [χ^2^(3) = 8.63; *p* = 0.035], with significant differences between trials with alcoholic versus nonalcoholic primes under placebo treatment only (nalmefene: *Z* = −1.03, *p* = 0.304; placebo: *Z* = −3.58, *p* < 0.001; **Figure 4B**). Treatment effects were not significant for either prime type (alcoholic: *Z* = −0.17, *p* = 0.862; nonalcoholic: *Z* = −0.34, *p* = 0.732). In order to avoid the fallacy of interpreting the difference between statistically significant and nonsignificant effects as “significant” (Gelman and Stern, 2006), we calculated nonalcoholic–alcoholic RT differences for each patient and performed an additional Wilcoxon signed-rank test to assess if the effect of nalmefene on reducing alcoholic cue-induced response invigoration was statistically meaningful. Indeed, we found significant differences between data from the placebo and the nalmefene sessions (*Z* = −2.20, *p* = 0.028; **Figure 4C**), indicating that prime type was not influencing the speed of responding after active treatment.

#### Event-Related Potentials

##### Occipital P1, N1, and P2 Amplitudes

**Figure 5:**
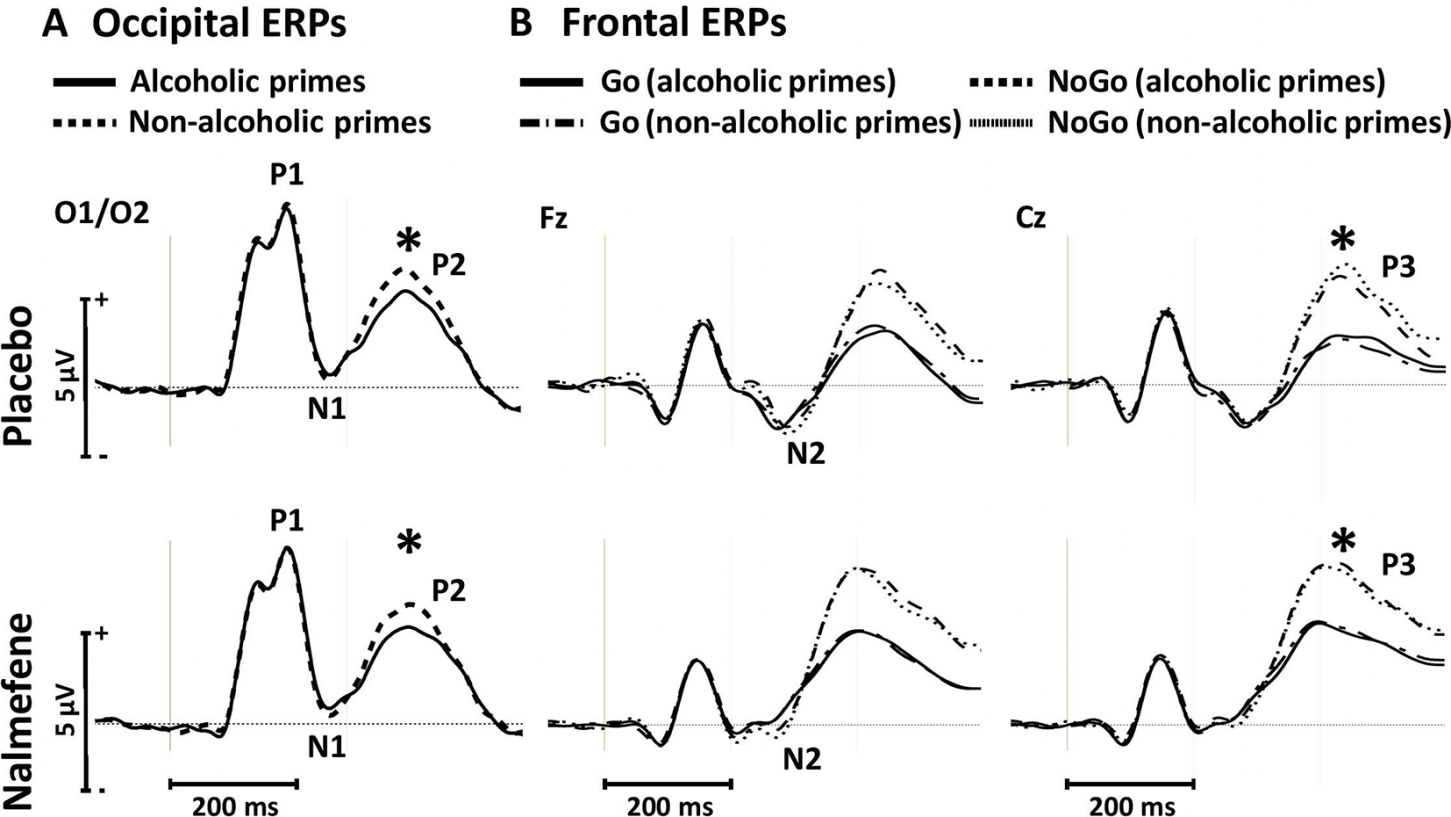
Occipital event-related potentials evoked by alcoholic and nonalcoholic primes **(A)** and frontal event-related potentials evoked by Go and NoGo targets in the context of alcoholic and nonalcoholic primes **(B)** in experiment 2. Stars depict significant (*p* < 0.05) differences between amplitudes obtained in different conditions.

For the P1 and N1 amplitudes, we found no significant main effects or interactions (**Figure 5A**, **Table 3**). Similar to the effect observed in experiment 1, P2 amplitudes were sensitive to prime type, with smaller P2 amplitudes for alcoholic cues, but importantly, this effect was not modified by treatment type (**Table 3**).

**Table 3:** Statistical results (main effects and interactions) of ANOVA performed for the occipital P1, N1 and P2 components in Experiment 2.

ERP	Main effects and interactions	df	F	*p*	η_p_^2^
**P1**	MEDICATION	1,18	1.094	0.310	0.057
	PRIME	1,18	0.044	0.836	0.002
	MEDICATION x PRIME	1,18	0.680	0.420	0.036
**N1**	MEDICATION	1,18	0.001	0.973	0.000
	PRIME	1,18	0.912	0.352	0.048
	MEDICATION x PRIME	1,18	0.225	0.641	0.012
**P2**	MEDICATION	1,18	0.044	0.836	0.002
	PRIME	1,18	15.476	0.001*	0.462
	MEDICATION x PRIME	1,18	0.069	0.796	0.004

*p < 0.01; ERP, Event Related Potentials.

##### Frontal N2 and P3 Amplitudes

Grand averaged frontal waveforms are presented in **Figure 5B**, with the corresponding statistical results shown in **Table 4**. For N2 amplitudes, no significant main effects or interactions were found. For the P3 component, repeated-measures ANOVA revealed larger P3 amplitudes for NoGo stimuli, but again, this component was insensitive to the administration of nalmefene or to the type of priming (**Table 4**).

**Table 4:** Statistical results (main effects and interactions) of ANOVA performed for the frontal N2 and P3 components in experiment 2.

ERP	Main effects and interactions	df	F	*p*	η_p_^2^
**N2**	MEDICATION	1,18	0.095	0.761	0.005
	PRIME	1,18	0.035	0.854	0.002
	TARGET	1,18	0.304	0.588	0.017
	MEDICATION x PRIME	1,18	0.521	0.480	0.028
	MEDICATION x TARGET	1,18	0.628	0.438	0.034
	PRIME x TARGET	1,18	0.093	0.764	0.005
	MEDICATION x PRIME x TARGET	1,18	1.467	0.241	0.075
**P3**	MEDICATION	1,18	0.632	0.437	0.034
	PRIME	1,18	0.143	0.710	0.008
	TARGET	1,18	19.452	< 0.001*	0.519
	MEDICATION x PRIME	1,18	0.000	0.985	0.000
	MEDICATION x TARGET	1,18	0.000	0.985	0.000
	PRIME x TARGET	1,18	1.387	0.254	0.072
	MEDICATION x PRIME x TARGET	1,18	0.016	0.900	0.001

*p < 0.01; ERP, Event Related Potentials*.*

## Discussion

In the current study, we aimed to investigate whether nalmefene influences the behavioral and neurophysiological correlates of appetitive cue processing, conflict detection, and response inhibition in AUD. A modified Go/NoGo paradigm was used, and ERPs were analyzed above occipital and frontal brain regions to assess the effect of treatment, alcoholic cues, and NoGo stimuli on cortical mechanisms of visual processing and executive control. Below, we shall discuss our results, starting with the behavioral effects of nalmefene.

### Nalmefene Mildly Reduces Response Vigor to Alcoholic Cues in Patients With AUD

With respect to our behavioral results, rarely occurring NoGo stimuli were associated with higher rates of erroneous responses relative to Go stimuli in both experiments, but this effect was not influenced by nalmefene in AUD patients, and neither did we found nalmefene versus placebo differences in terms of the sensory index (*d*′). Although we did not directly compare behavioral measures from our healthy group to patient data from the placebo session (for reasons discussed below, under “Limitations”), the performance of both groups was strikingly similar, with near-ceiling effects (**Table 2**). Comparable behavioral performance on Go/NoGo tasks in patients with AUD relative to healthy controls has been commonly reported in the literature (Luijten et al., 2014), but it is puzzling why we did not detect more false alarms and lower *d*′ values in alcohol-primed trials in placebo-treated patients. Impaired executive control in the context of alcohol cues has been well documented in social drinkers (Martinovic et al., 2014; Dyke and Fillmore, 2015), but in our study, such effects might have been masked by the very high accuracy rates in the patient group. Perhaps, using shorter (< 1,000 ms) time windows for responding would have been more appropriate for uncovering between-treatment differences in accuracy or sensitivity index measures.

The main finding of our study is that primes depicting pictures of alcoholic drinks were followed by significantly faster response times in patients receiving placebo, while such an effect was absent in patients receiving nalmefene and in healthy participants. Despite this statistically significant effect, it is important to highlight that the magnitude of the RT difference between the two treatment conditions was very small (in the range of few milliseconds; **Figure 4C**), rendering the current finding behaviorally and clinically less pronounced. Although we consider the RT effect to be very small in our study, it is primarily due to the weak influence of alcoholic cues on response times under placebo, and thus, our finding does not preclude the possibility that nalmefene could have exerted more robust effects under different circumstances. Namely, it is possible that either by recruiting a patient group that is more sensitive to our alcoholic cues (e.g., by investigating nonabstinent patients) or by modifying our paradigm to be more sensitive to detect cue-induced response invigoration in our patient sample (e.g., using a shorter time window for responding), we would have observed nalmefene versus placebo differences that are behaviorally also meaningful. The fact that even alcoholic cues induced substantially longer responses in AUD patients than in healthy participants (**Figures 2B** vs. **4B**) points toward a general psychomotor slowing in AUD, which is well-known in this disorder (De Wilde et al., 2007; Lawrence et al., 2009).

Another interesting aspect of our result is that nalmefene did not prolong reaction times in general, which is in contrast to the finding reported by Quelch et al. (2017). While the reason for the absence of an overall RT prolongation after active treatment in our study is unclear, we speculate that it might be related to differences in time constraints. In particular, patients were provided with plenty of time (1,000 ms) to respond to targets in the current paradigm, whereas they had to react within a much shorter time window (150–300 ms) in the study by Quelch et al. (2017). Taken together, our result indicates that alcoholic stimuli might facilitate approach tendencies in AUD, an effect that is likely to be governed by learned Pavlovian-type associations and has been associated with the dopaminergic system (Everitt and Robbins, 2005; Garbusow et al., 2014). Indeed, fast reaction times (a measure of response vigor) were linked to phasic dopamine signals (Satoh et al., 2003), and considering that appetitive cues in our paradigm might have enhanced the firing of midbrain dopaminergic neurons in patients with placebo, our finding of faster responses upon alcoholic primes fits well into the incentive-sensitization theory of addiction (Robinson and Berridge, 2003). The fact that such response invigoration for alcoholic primes disappeared after nalmefene intake indicates that nalmefene might have inhibited subcortical dopaminergic transmission. Although we argue that this molecular effect of nalmefene is related to modulations of the Pavlovian learning system in patients, we note that our Go/NoGo task was substantially different from PIT tasks that have been successfully used to elucidate the nature of Pavlovian response biases in AUD (Garbusow et al., 2014; Schad et al., 2019). In particular, our task did not involve a conditioning phase or instrumental learning whatsoever, and therefore, it might be sensitive to psychological processes and neural circuits distinct from those associated with PIT tasks.

### Nalmefene Does not Influence Neural Responses to Alcohol Cues or ERPs Reflecting Executive Control

With respect to prime-induced early visual ERPs, no effects were found for the posterior P1 and N1 components, suggesting that these ERPs were not influenced by prime type (alcoholic vs. nonalcoholic) or treatment (nalmefene vs. placebo). On the other hand, the P2 component was significantly larger for nonalcoholic relative to alcoholic primes in both experiments. The posterior P2 not only has been implicated in perceptual grouping and attention (Han et al., 2005; Straube and Fahle, 2009; Schendan and Lucia, 2010), but it is also sensitive to emotional valence of visual images, being larger in amplitude for unpleasant stimuli (Carretié et al., 2001; Olofsson and Polich, 2007). Thus, there is no clear functional description of the visual P2, and given that our nonalcoholic primes depicted popular drinks commonly associated with positive (rather than negative) feelings, our results regarding the P2 are difficult to interpret. Still, it is clear that nalmefene did not modulate this component, and therefore, we can conclude that all three visual ERPs were insensitive to our pharmacological intervention. This was a surprising result since these posterior components reflect not only bottom–up sensory processing of visual inputs but are also sensitive to top–down influences such as the effects of attention, expectation, and emotional responses (Carretié et al., 2001; Han et al., 2005; Straube and Fahle, 2009; Schendan and Lucia, 2010). Thus, we hypothesized that, through direct and indirect effects of nalmefene on opioid and dopaminergic signaling, this drug would alter the sensitivity of visual ERPs to appetitive and more salient cues depicting alcoholic drinks. The fact that such an effect was absent from our EEG data despite observing cue-induced response invigoration on the behavioral level points toward subcortical pharmacological effects of nalmefene that rendered early cortical analysis of visual stimuli intact.

We also analyzed the target-locked frontal N2 and P3 components that reflect conflict monitoring and inhibitory processes (Huster et al., 2011; Luijten et al., 2014), and therefore, they can be regarded as neural indices of efficient executive control in our task. We found that only healthy participants showed larger NoGo-associated N2 amplitudes, pointing towards impaired conflict detection for NoGo trials in the patient group. The magnitude of NoGo versus Go amplitude differences for the N2 component has been previously associated with the degree of alcohol avoidance in a subclinical sample, such that larger NoGo-related N2 amplitudes were found in individuals with high avoidance from alcohol (Kreusch et al., 2014). Although we did not collect data on the predisposition to refrain from alcohol consumption, our result point towards low alcohol avoidance in our patient group, which is not surprising given that they were all abstinent for <2 months at the time of data collection. Our finding on the N2 also aligns well with that reported by Pandey and colleagues (Pandey et al., 2012) and strengthen views on impaired executive control and frontal lobe (in particular, dACC) dysfunction in AUD (Moselhy et al., 2001; Ratti et al., 2002; Kamarajan et al., 2005; Pandey et al., 2012).

In line with the vast amount of literature on the ERP correlates of response inhibition (Huster et al., 2011; Luijten et al., 2014; Hong et al., 2017), we observed larger central P3 amplitudes for NoGo stimuli in both experiments and for both treatment sessions in patients. These findings indicate that cortical processes associated with inhibiting automatic response tendencies were intact in our patients. Previous studies focusing on the P3 in tasks requiring inhibitory control in AUD yielded mixed results, reporting either reduced or intact P3 amplitudes in patients (Luijten et al., 2014). As for the absence of the NoGo-related N2 effect, the large NoGo versus Go P3 amplitude difference in our patients might be related to their low alcohol avoidance (Kreusch et al., 2014), but this should have been assessed explicitly in the current study. Given that the P3 is the last prominent ERP in the cascade of neural events associated with response inhibition, and it has been localized to cortical regions such as the supplementary motor area (Huster et al., 2010; Huster et al., 2011), we can expect that its amplitude is informative of the success of motor inhibition. Indeed, in parallel to the significant Go versus NoGo P3 amplitude difference in our patient group that was insensitive to nalmefene, we did not find differences in response accuracy or the sensitivity index after nalmefene versus placebo intake. Overall, we conclude that deficits in conflict monitoring (N2 component) and intact neural correlates of motor inhibition (P3 component) in AUD are (I) not modified by alcohol cues and (II) are not influenced by nalmefene. Thus, it is not likely that any of these ERPs are related to the beneficial behavioral effect of nalmefene on alcohol-primed RTs.

To our knowledge, the study by Quelch and colleagues (Quelch et al., 2017) was the only study that investigated the neural effects of nalmefene in human participants using neuroimaging methods. Although the authors found reduced blood-oxygen-level-dependent responses following nalmefene administration in the striatum and brainstem but not in any cortical area, regions in the frontal cortex (such as the dACC) that are interconnected with these dopaminergic structures (Di Martino et al., 2008; Furman et al., 2011) could have been theoretically influenced by neurochemical alterations caused by the drug. Based on this, we hypothesized that the effect of nalmefene would manifest in enhanced N2/P3 amplitudes in a task that relies on conflict monitoring and response inhibition. However, our null results in this regard support the functional magnetic resonance imaging findings of Quelch and colleagues (Quelch et al., 2017) in that nalmefene might primarily act *via* subcortical mechanisms in AUD and does not influence cortical activity arising from multiple sources in the frontal lobe that have been implicated in executive control. This conclusion coincides with nalmefene’s clinical indications: this agent is primarily used as an as-needed, harm-reducing treatment option rather than as therapy for reaching long-term abstinence.

### Limitations

Although participants from experiment 1 did not differ in gender distribution, age, and education level from patients enrolled in experiment 2 [gender: χ^2^(1) = 0.009, *p* = 0.925; age: *t*(37) = 1.695, *p* = 0.098; education: *U* = 166, *Z* = −0.76, *p* = 0.447], the main limitation of our study is that we could not directly compare data from healthy participants to those collected in patients receiving placebo for three reasons. First, our healthy participants were not blind to the treatment, as they were informed about receiving placebo. Second, the crossover design of experiment 2 might have induced learning effects for the placebo data (i.e., half of patients started with the nalmefene session), making the control versus patient comparison problematic. Third, we had to use a different EEG device in experiment 2, which might have introduced latent effects to the ERPs. Therefore, we made only qualitative between-group comparisons for two relevant measures (longer RTs in patients, the absence of the Go vs. NoGo effect for the N2 component in patients), and these should be treated with caution.

Another limitation is that, due to the strict ethical restrictions about the administration of nalmefene to healthy adults, we could not investigate the behavioral and neural effects of this agent in individuals without AUD. Finally, our patient group was heterogeneous in terms of the duration of abstinence (i.e., between 2 weeks and 2 months), and given that the patients at different stages of abstinence from substance abuse show distinct patterns of cognitive impairment (Fernández-Serrano et al., 2011), it is possible that different outcomes could have been observed in a more homogenous sample.

### Conclusions

This work is one among the few studies (Quelch et al., 2017) designed with the aim to fulfil the informational gap in understanding how nalmefene influences cortical mechanisms associated with executive control and the processing of alcoholic cues. It seems that this agent affects approach behavior when patients with AUD are primed with pictures depicting alcoholic drinks, but this effect was very mild in our study, with limited behavioral relevance. Moreover, we found that nalmefene does not influence ERP signatures of executive control or visual processing. These results suggest that nalmefene modulates response invigoration presumably related to subcortical dopaminergic transmission, as it did not influence cortical processing in our task. Future studies could design experimental paradigms that enable exploring the mechanisms of nalmefene’s effect on incentive salience more precisely.

## Data Availability

All materials and anonymized data are available upon request.

## Ethics Statement

The studies involving human participants were reviewed and approved by Clinical Research Coordination Centre, University of Szeged. The patients/participants provided their written informed consent to participate in this study.

## Author Contributions

BG and TK performed data collection with the help of PÁ, AA, and ID. The study was designed by GC and PÁ. Data analysis was performed by GC, BG, and TK. The manuscript was prepared by GC, PÁ, BG, and KH; this was further reviewed by the coauthors. ZJ contributed expert opinion to the manuscript correction.

## Funding

Funding for this study was provided by the University of Szeged; no external sources were involved. The authors decided on study design, data management, writing the paper, and submitting it for publication.

## Conflict of Interest Statement

PÁ and ZJ were ex-consultants of Lundbeck on independent research area from addictology.

The remaining authors declare that the research was conducted in the absence of any commercial or financial relationships that could be construed as a potential conflict of interest.
